# Practices of maternity nurses regarding perinatal bereavement care: a cross-sectional study

**DOI:** 10.1186/s12884-025-08563-3

**Published:** 2025-12-27

**Authors:** Nora Monier Elsaka, Abdelaziz Elrefaeey, Eman Sameh AbdElhay, Samia I Hassan, Hanan Elsayed Mohamed Elsayed

**Affiliations:** 1 Woman’s Health and Midwifery Nursing, Faculty of Nursing, Mansoura University, Dakahlia Governorate, Mansoura City, Egypt; 2 Obstetrics and Gynecology, Faculty of Medicine, Mansoura University, Dakahlia Governorate, Mansoura City, Egypt; 3 Psychiatric Nursing and Mental Health, Faculty of Nursing, Mansoura University, Dakahlia Governorate, Mansoura City, Egypt

**Keywords:** Grief, Nurses, Practices, Perinatal bereavement care, Perinatal loss

## Abstract

**Background:**

Pregnancy is often considered the happiest experience in a woman’s life. However, perinatal loss may sadly occur as one of its possible outcomes, which necessitates the provision of bereavement care by skilled nurses. Consequently, this study aimed to assess the practices of maternity nurses in terms of perinatal bereavement care.

**Methods:**

A descriptive cross-sectional study design, which utilized a convenient sample of 117 maternity nurses who worked at Mansoura University Hospital (MUH), Egypt, was used. Data were collected throughout February to April 2025 via a structured interview questionnaire, which consisted of two parts, including the general and professional characteristics of maternity nurses and maternity nurses’ self- reported practices regarding perinatal bereavement care. Pearson’s chi-square test (x²) was used, the Fisher exact test (FE) was used when possible.

**Results:**

The study findings revealed that 73.5% of the studied maternity nurses had a satisfactory level of practice regarding perinatal bereavement care. Notably, 0.0% of them used the partogram during labor. Furthermore, only 17.1% of them allowed skin-to-skin contact with the dead baby.

**Conclusion:**

Although maternity nurses generally reported a satisfactory level of practice regarding perinatal bereavement care, some deficiencies were reported during the intrapartum period.

## Introduction

Gestation is a process of changes that take place in a woman’s body organs and systems due to the growth of the foetus [1]. Birth outcomes differ from one pregnancy to another and may include live birth, stillbirth, abortion, miscarriage, and early neonatal death [2]. Newborn death within the first 7 days of life and stillbirth (loss from 22 weeks of pregnancy with a birth weight over 500 g) are included in the World Health Organization’s definition of perinatal loss. However, the literature tends to broadened this meaning to include any unintentional loss up to 28 days after delivery included spontaneous abortion, stillbirth, or neonatal death [3].

Perinatal loss influences one in ten women globally and has a significant effect on both the mental and physical health of bereaved women [4]. The global stillbirth rate (per 1000 total births) in 2023 was 14.3 [13.7–15.5], whereas the stillbirth rate in Egypt was 8.4 [4.8–14.7] in the same year [5]. Approximately 23 million miscarriages occur globally each year, which means that approximately 44 pregnancy losses occur every minute. On average, the risk of miscarriage affects 15.3% of all recognized pregnancies, according to combined results from several studies (with a 95% confidence interval of 12.5% to 18.7%) [6].

Moreover, perinatal loss is linked to increased healthcare and societal expenditures because of the detrimental psychological effects on parents, including depression, anxiety, posttraumatic stress disorder, grief, psychosis, and suicide attempts, which lead to poor physical health, a decline in financial standing, and a loss of job. It also signifies a main loss of parental status as well as secondary losses, including a loss of hope, security, and child-rearing experience [7, 8]. Furthermore, it is associated with a greater risk of metabolic diseases, cardiac disease, and persistent pain [9].

The perinatal bereavement care concept includes comprehensive care for mental, emotional, physical, and spiritual support, which healthcare professionals offer to couples and family members during the perinatal bereavement experience [10]. Nurses and midwives play a crucial role in offering support, education, and treatment options to women suffering from perinatal loss. When bereaved families are exposed to the similarity between the suspected care and the care that is already given to them, the severity of their bereavement decreases [11].

High-quality grief care focuses on offering couples chances to interact with their deceased baby and make memories, such as holding the infant, taking pictures, and participating in memorial services. A more adaptable grieving process and several better long-term psychosocial outcomes have been linked to these practices of care [12]. However, there are few data on bereavement care and perinatal loss practices in most settings, particularly in middle- and low-income areas where the strain is highest [13]. Additionally, there is limited research on the practices of maternity nurses regarding perinatal bereavement care in Egypt, especially in Mansoura city. Therefore, this study was conducted with the aim of assessing the practices of maternity nurses in terms of perinatal bereavement care.

### Research question

What are the maternity nurses’ practices regarding perinatal bereavement care?

## Methods

### Research design

A descriptive cross-sectional study design was used to accomplish the study’s aim. This type of observational research design in which variables and related data are observed and measured simultaneously by the researcher. Every subject received a single observation, and no follow-up was conducted; instead, the variables were measured at the time of the observation [14]. Data were collected through face-to-face structured interviews with participants, not through direct observation of their clinical practice. Nurses were interviewed about their routine practices when providing care to women experiencing perinatal loss, based on their professional experience. The interview did not focus on any single recent bereavement event.

### Study setting

The current study was conducted at the obstetrics and gynecology inpatient wards (ward numbers: 9, 10, 15 & 18), the labor & delivery unit, the obstetric critical care unit, and the obstetric emergency unit at Mansoura University Hospital, Mansoura city, Dakahlia Governorate, Egypt. MUH is affiliated with the Ministry of Higher Education, which provides free services for women during the antepartum, intrapartum, and postpartum periods in addition to gynecological services. Mansoura city is the capital of Dakahlia Governate in the Nile Delta region, with a population of approximately 630,000. MUH is a tertiary teaching hospital providing specialized obstetrics care. As a university hospital, it serves as a referral center for complicated pregnancies and high-risk cases from surrounding districts.

### Study participants

This study employed a convenience sample comprising 117 maternity nurses who work in the previously described setting and were present at the time of data collection. The researcher excluded maternity nurses who carried out administrative duties and nurses who had less than six months of experience.

### Sample size calculation

On the basis of data from the study Kiu et al. [15], where 58.8% of participants practice favourable caring behaviours regarding perinatal bereavement care. The sample size was calculated with a precision/absolute error of 5% and a type 1 error of 5%, according to the following formula:

$$\begin{aligned} N&=\frac{p_0q_0\left\{Z_{1-\alpha/2}+Z_{1-\beta}\sqrt{\displaystyle\frac{p_1q_1}{p_0q_0}}\right\}^2}{\left(p_1-p_0\right)^2}\\q_o&=1-p_0\\q_1&=1-p_0\\N&=\frac{0.588\ast0.412\left\{1.96+0.84\sqrt{\displaystyle\frac{0.46\ast0.54}{0.588\ast0.412}}\right\}^2}{\left(0.46-0.588\right)^2}\\N&=117\end{aligned}$$

where p_0_ = proportion (incidence) of the population, p_1_ = proportion (incidence) of the study group, N = sample size for the study group, and α = probability of type I error (usually 0.05).

β = probability of type II error (usually 0.2), z = critical Z value for a given α or β.

On the basis of the above formula, the total sample size required for the study was 117 maternity nurses.

### A tool for data collection

One tool was utilized for data collection:

### A structured interview questionnaire

It was divided into two parts:

- Part 1: General and professional characteristics of maternity nurses. It consists of 11 items, such as age, level of education, years of experience, and marital status.
- Part 2: Maternity nurses’ self -reported practices regarding perinatal bereavement care. The researcher developed it after reviewing the related literature [16–18]. It consists of 4 domains, including:
- Domain (I): Maternity nurses' reported practices regarding perinatal bereavement care during the admission period; this domain consisted of 13 questions, such as providing verbal and nonverbal information regarding diagnosis and treatment sensitively and obtaining past obstetric and medical history on admission. Domain (II): Maternity nurses’ reported practices regarding perinatal bereavement care during the intrapartum period; this domain consisted of 9 questions, such as administering analgesics to bereaved women during delivery and offering reassurance to mothers during labor/delivery. Domain (III): Maternity nurses’ reported practices regarding perinatal bereavement care during the postpartum period; it consisted of 7 questions, such as providing information regarding what to do with the expelled product of conception and providing medication to suppress lactation during the postpartum period. Domain (IV): Maternity nurses’ reported practices regarding perinatal bereavement care during the discharge period; this domain consisted of 7 questions, such as providing information about when to conceive again and providing parents with clear verbal and written details of the process for follow-up appointments.

### Scoring system

Each question has two alternative answers: yes and no. The responder was scored (one) for yes and (zero) for no for each response. The total score for each nurse was calculated by summing all items (36 items total across the four domains). This total score was then converted to a percentage by dividing the nurse’s score by the total possible score (36) and multiplying by 100. A total percentage score of less than 60% was classified as unsatisfactory practice, whereas a percentage score of 60% or higher was considered satisfactory practice [19].

### Tool validity and reliability

Five experts represented diverse relevant specializations: woman’s health and midwifery nursing, obstetrics and gynecology, and psychiatric nursing and mental health, ensuring comprehensive coverage of the subject matter. An initial pool of 45 items. After evaluation, 9 items were removed due to redundancy or lack of relevance, resulting in the final 36-item tool. Content Validity Index (CVI) was not formally calculated as this was a formative expert review process rather than quantitative content validation. Experts focused on clarity, relevance, and clinical applicability. After certain changes were made (such as making some questions easier and reorganizing their sequence), the final form was utilized to gather data. The tool was then pilot tested with 12 maternity nurses to identify any items that needed to be reworded to facilitate comprehension in the specific characteristics setting being addressed. The analysis of the pilot results indicated that the statements in the questionnaire were understandable, relevant, and clear to the study subjects and that no modifications were applied. Therefore, the pilot study sample was included in the total sample at the time of data analysis. This step was completed at the end of January 2025. High reliability was demonstrated by the internal consistency (Cronbach’s alpha) of the study tool, which was 0.778.

### Data collection process

The process was structured into two main phases: the preparatory phase and the operating phase.

### Preparatory phase

During this phase, the researcher secured official approval from the Head of the Woman’s Health and Midwifery Nursing Department and the Vice Dean for Postgraduate Studies and Research at the Faculty of Nursing at Mansoura University. An official letter was subsequently sent to the Head of the Obstetrics and Gynecology Department at MUH following a clear explanation of the study’s aim and nature, upon which official permission to conduct the study was granted. The data collection tool was initially developed in English and subsequently translated into Arabic, ensuring cultural and contextual appropriateness. 10% of the sample size was used in a pilot study prior to the actual sample being gathered.

### Operating phase

I. Data collection phase
  The study’s actual fieldwork begins in early February 2025 and lasts until the end of April 2025. Three days a week (Saturday, Monday, and Wednesday) from 9 a.m. to 3 p.m., the researcher was present in the previously described setting on these days, as maternity nurses had available time for data collection.
  The researcher introduced herself to the maternity nurses, informed them about the study’s aim, and ensured that the maternity nursing staff fulfilled all the criteria of the study. Once data confidentiality was confirmed, they gave their written consent to participate in the study, and they could withdraw from the current study at any time.
  Each studied maternity nurse was interviewed separately by the researcher for 20–30 min. Nurses were asked to report their practices regarding perinatal bereavement care through structured interview questions. This represents self-reported practices, not observed practices.
  The researcher collected data from the studied maternity nurses, with an average of 3 nurses on each of the previously mentioned days of the data collection period.
  Until the sample size was reached, the researcher attended the previously described setting.
II. Data analysis phase
  The collected data were saved, organized, categorized, coded, transferred into specially created formats, and then statistically analysed via the SPSS program version 25. The normality of the practice score was assessed using the Shapiro-Wilk test, which indicated significant deviation from normal distribution (W = 0.934, *p* = 0.000). Categorical data are presented as numbers and percentages. Relationships between practice level and other categorical variables were examined using Pearson's Chi-square test (χ²) and Fisher's exact test (FE) was used, when possible. The level of statistical significance was set at a p value ≤ 0.05, and results with a *p* value ≤ 0.001 were considered highly significant.

## Results

Table 1 shows that 34.2% of the studied maternity nurses had ages ranging from 25 to less than 30 years with a mean age ± SD(28.48 ± 5.58), 69.2% of participants graduated from the technical institute of nursing, 49.6% of the study sample had 6 months to less than 5 years of experience, 68.3% of participants were married, 21.4% had personal perinatal bereavement experience, and 82.9% of participants faced10 or more bereaved parents while they were working.

**Table 1:** Distribution of the studied maternity nurses according to their general and professional characteristics (*n* = 117)

Variable	*n* (117)	%
Age in years		
20 - < 25 years	37	31.6
25 - <30 years	40	34.2
30 - <35 years	23	19.7
35 - <40 years	11	9.4
≥ 40 years	6	5.1
Mean ± SD (Range)	28.48 ± 5.58 (21–45)	
Level of education		
Diploma of Nursing	18	15.4
Technical institute of nursing	81	69.2
Bachelor of Nursing and above	18	15.4
Years of experience		
6 months - <5 years	58	49.6
5 - <10 years	23	19.7
10 - <15 years	19	16.2
≥ 15 years	17	14.5
Mean ± SD (Range)	7.18 ± 6.08 (1–26)	
Marital status		
Single	36	30.8
Married	80	68.3
Divorced	1	0.9
Personal perinatal bereavement experience		
No	92	78.6
Yes	25	21.4
The total number of circumstances involving bereaved parents		
˂ 5 times	11	9.4
5 -< 10 times	9	7.7
≥ 10 times	97	82.9

**Fig. 1:**
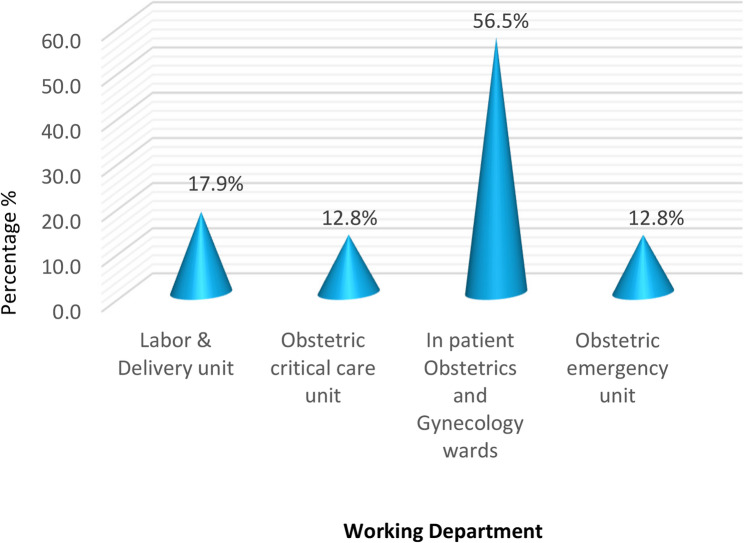
Distribution of the studied maternity nurses according to their working department (*n*=117)

Figure 1 illustrates that 56.5% of the studied maternity nurses worked in inpatient obstetrics and gynecology wards, 17.9% worked in labor and delivery unit, 12.8% worked in obstetric critical care unit, and 12.8% worked in obstetric emergency unit.

Table 2 shows that 59.8% of the studied maternity nurses had not received any training related to bereavement care. Among the 40.2% who had received training, 40.4% received it only once.

**Table 2:** Distribution of the studied maternity nurses according to their previous training related to bereavement care (*n* = 117)

Variable	*n* (117)	%
Previous training related to bereavement care		
No	70	59.8
Yes	47	40.2
Number of training received for provision of bereavement care (*n* = 47) %		
Once	19	40.4
2 times	10	21.3
3 times or more	18	38.3
The last training about provision of bereavement care (*n* = 47) %		
< 6 months	19	40.4
6 - < 12 months	9	19.2
≥ 12 months	19	40.4

**Fig. 2:**
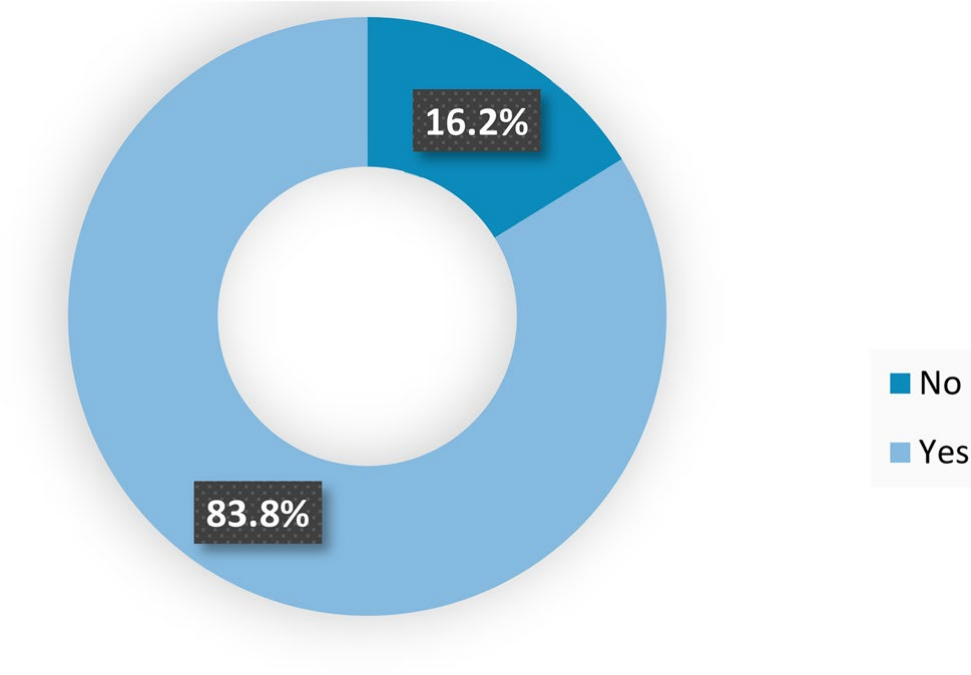
Presence of a clear policy for the management of bereaved women in the working department (*n*=117)

Figure 2 illustrates that 83.8% of the studied maternity nurses reported the presence of a clear policy for the management of bereaved women in their working department. In comparison, 16.2% of participants reported that there was no clear policy for the management of bereaved women in their working department.

Table 3 shows that most (95.7%) of the studied maternity nurses requested Rhesus factor (Rh), complete blood count (CBC), and blood clotting tests. Additionally, the majority of participants (82.1% and 87.2%, respectively) offer individualized preparations for delivery and request an obstetric ultrasound to confirm intrauterine foetal death (IUFD). Moreover, approximately two-thirds (65.8%) of participants obtained written consent for any invasive procedure on the baby. However, 77.8% of the study sample did not ask bereaved women about their preference for the mode of delivery. Additionally, more than half (63.2%) of participants did not accommodate bereaved women away from other cases of normal pregnancy.

**Table 3:** Distribution of the studied maternity nurses according to their reported practices regarding perinatal bereavement care during the admission period (*n* = 117)

Practices during the admission period	No	Yes
	*n* (%)	*n* (%)
Welcome the bereaved woman to the ward and provide orientation to the room, call bell, and facilities	14 (12.0)	103 (88.0)
Give the bereaved woman a direct admission card with an explanation	23 (19.7)	94 (80.3)
Accommodate a woman who was admitted to the hospital with a diagnosis of perinatal loss, away from other cases of normal pregnancy	74 (63.2)	43 (36.8)
Scheduling the bereaved woman’s appointment to be the first woman seen by the sonographer and obstetrician on that day	53 (45.3)	64 (54.7)
Providing verbal and nonverbal information regarding diagnosis and treatment in a sensitive manner	19 (16.2)	98 (83.8)
Obtaining past obstetric and medical history on admission	22 (18.8)	95 (81.2)
Offering individualized preparation for delivery tailored to women’s needs and health conditions	21 (17.9)	96 (82.1)
Preparing parents for the appearance of a baby after birth	84 (71.8)	33 (28.2)
Requesting an obstetric ultrasound to confirm IUFD before the decision of delivery	15 (12.8)	102 (87.2)
Requesting Rh, CBC, and blood clotting tests on admission	5 (4.3)	112 (95.7)
Asking bereaved women about their preference for the mode of delivery and explaining their preference	91 (77.8)	26 (22.2)
Obtaining written consent for any invasive procedure on the baby, including tissues taken for genetic analysis	40 (34.2)	77 (65.8)
Offering pain relief measures according to hospital policy	75 (64.1)	42 (35.9)

Table 4 shows that most (91.5%) of the studied maternity nurses offered reassurance to mothers during labor/delivery. Additionally, 65.8% of the study sample monitored maternal conditions for women with stillbirth as frequently as those with a live intrauterine baby. However, 66.7%, 100.0%, 74.4% and 82.9% of participants did not allow bereaved women to deliver in separate rooms; did not use a partogram; did not administer analgesics; and did not allow skin-to-skin contact with the dead baby.

**Table 4:** Distribution of the studied maternity nurses according to their reported practices regarding perinatal bereavement care during the intrapartum period (*n* = 117)

Practices during the intrapartum period	No	Yes
	*n* (%)	*n* (%)
Allowing bereaved women to deliver in a separate room from others with live intrauterine pregnancies	78 (66.7)	39 (33.3)
Offering reassurance to mothers during labor/delivery	10 (8.5)	107 (91.5)
Send cord blood samples if Rh negative for clinical laboratory and Placental tissue for cytogenetic testing	48 (41.0)	69 (59.0)
Monitoring maternal condition for women with stillbirth is as frequent as that for women with live intrauterine babies	40 (34.2)	77 (65.8)
Using the partogram during labor	117 (100.0)	0 (0.0)
Administering analgesics to bereaved women during delivery	87 (74.4)	30 (25.6)
Asking all parents whether they have any religious, cultural, or spiritual needs and facilitating requests where it is possible to alleviate pain, like listening to the Quran or music	67 (57.3)	50 (42.7)
Allowing skin-to-skin contact with a dead baby	97 (82.9)	20 (17.1)
Administering prophylactic intrapartum antibiotics for women suspected of chorioamnionitis	8 (6.8)	109 (93.2)

Table 5 shows that the majority (82.1%, and 88.0%, respectively) of the studied maternity nurses provided medication to suppress lactation and assessed bereaved women routinely for thromboprophylaxis. Moreover, 77.8% of participants provided information regarding what to do with the expelled product of conception. However, 42.7% of the study sample did not provide information regarding the death registration of a stillborn baby.

**Table 5:** Distribution of the studied maternity nurses according to their reported practices regarding perinatal bereavement care during the postpartum period (*n* = 117)

Practices during the postpartum period	No	Yes
	*n* (%)	*n* (%)
Offering a referral to another healthcare provider as a psychologist	27 (23.1)	90 (76.9)
Providing information regarding what to do with the expelled product of conception	26 (22.2)	91 (77.8)
Providing medication to suppress lactation during the postpartum period	21 (17.9)	96 (82.1)
Administering Anti-Rh D gamma globulin for Rh-negative women	21 (17.9)	96 (82.1)
Assessing bereaved women routinely for thromboprophylaxis	14 (12.0)	103 (88.0)
Providing information regarding death registration of stillbirth and advice to parents that the name of their baby after registration cannot be entered at a later date, nor can it be changed	50 (42.7)	67 (57.3)
Offering counselling to all bereaved women and their husbands	27 (23.1)	90 (76.9)

Table 6 shows that 94.9% of the studied maternity nurses provided recommendations about the importance of obstetric antenatal care in subsequent pregnancies. Moreover, 81.2% of participants provided information about when to conceive again. Additionally, 77.8% and 76.9% of the study sample provided information regarding coping styles and discussed the benefits of delaying conception until severe psychological issues were resolved.

**Table 6:** Distribution of the studied maternity nurses according to their reported practices regarding perinatal bereavement care during the discharge period (*n* = 117)

Practices during the discharge period	No	Yes
	*n* (%)	*n* (%)
Providing information regarding coping styles with the emotional responses of perinatal bereavement.	26 (22.2)	91 (77.8)
Providing information about when to conceive again.	22 (18.8)	95 (81.2)
Informing bereaved parents about the findings of the foetal tissue histopathology specimen.	19 (16.2)	98 (83.8)
Providing bereaved parents with clear verbal and written details of the process for follow-up appointments.	15 (12.8)	102 (87.2)
Discussing the potential benefit of delaying conception until severe psychological issues have been resolved.	27 (23.1)	90 (76.9)
Providing recommendations about the importance of obstetric antenatal care in subsequent pregnancies to prevent further loss.	6 (5.1)	111 (94.9)
Reviewing that the bereaved woman is given verbal and nonverbal information about her postnatal care and psychotherapy after discharge.	16 (13.7)	101 (86.3)

**Fig. 3:**
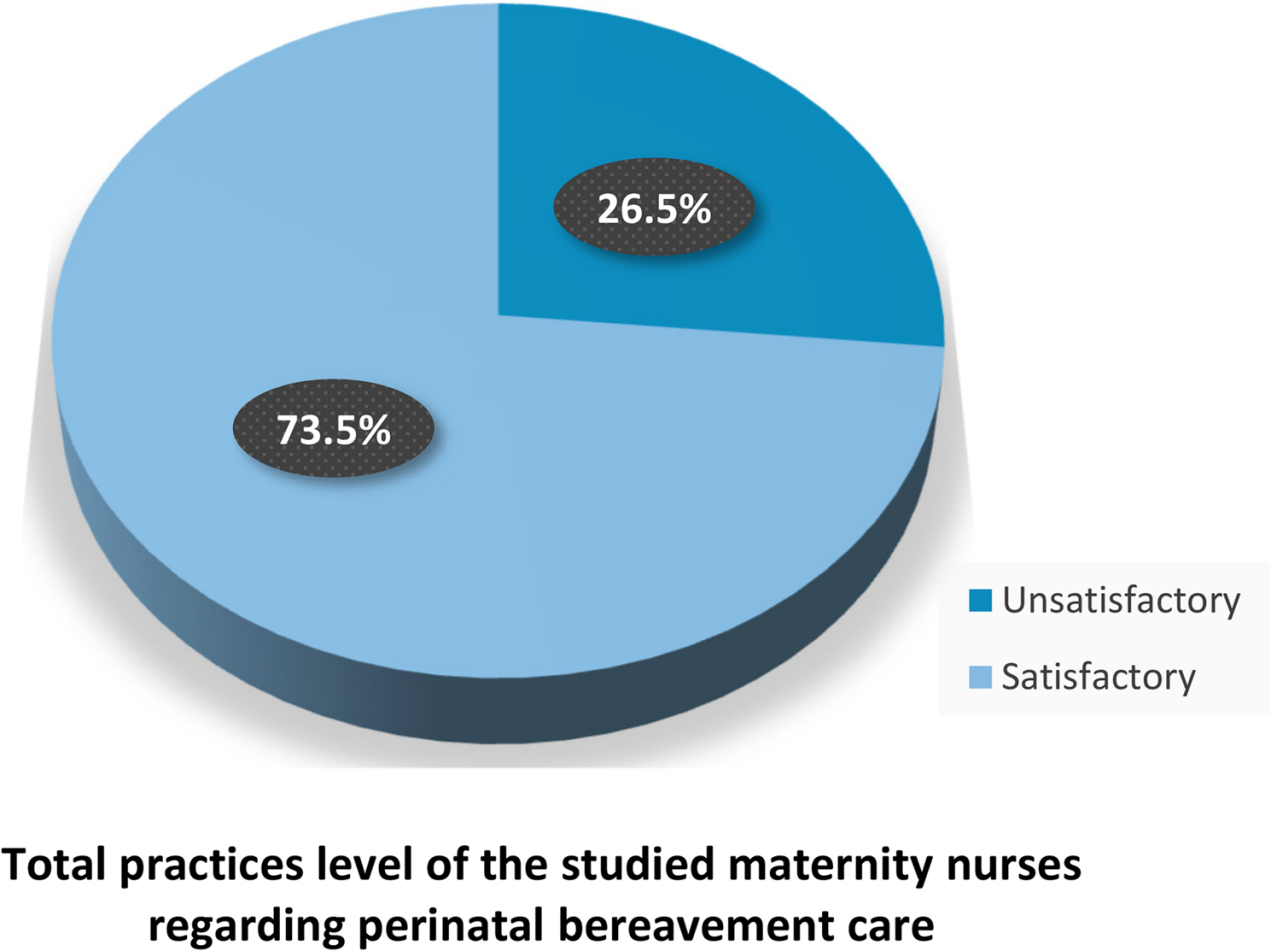
Distribution of the studied maternity nurses according to their total level of practices regarding perinatal bereavement care (*n*=117)

Figure 3 shows that 73.5% of the studied maternity nurses had a satisfactory level of practices regarding perinatal bereavement care, whereas only 26.5% had an unsatisfactory level of practices.

Table 7 shows that there was a highly statistically significant association between the studied maternity nurses’ working department and their reported level of practices regarding perinatal bereavement care (*P* = 0.000). There was no statistically significant association between their reported practice level and their age, level of education, or years of experience.

**Table 7:** Relationship between the general and professional characteristics of the studied maternity nurses and their reported level of practices regarding perinatal bereavement care (*n* = 117)

Variable	Practices level		Significance test	
	Unsatisfactory (*n* = 31)	Satisfactory (*n* = 86)	X^2^/FE	*p*
Age in years	*n* (%)	*n* (%)		
20 - < 25 years	15 (48.4)	22 (25.6)	9.281	0.540
25 - <30 years	6 (19.4)	34 (39.5)		
30 - <35 years	8 (25.8)	15 (17.4)		
35 - <40 years	1 (3.2)	10 (11.6)		
≥ 40 years	1 (3.2)	5 (5.8)		
Level of education				
Diploma of Nursing	5 (16.1)	13 (15.1)	0.044	0.978
Technical institute of nursing	21 (67.7)	60 (69.8)		
Bachelor of Nursing and above	5 (16.1)	13 (15.1)		
Years of experience				
6 months - <5 years	15 (48.4)	43 (50.0)	2.872	0.412
5 - <10 years	4 (12.9)	19 (22.1)		
10 - <15 years	5 (16.1)	14 (16.3)		
≥ 15 years	7 (22.6)	10 (11.6)		
Working department				
Labor & Delivery unit	4 (12.9)	17 (19.8)	39.691	0.000^***^
Obstetric critical care unit	3 (9.7)	12 (14.0)		
In patient Obstetrics and Gynecology wards	10 (32.3)	56 (65.1)		
Obstetric emergency unit	14 (45.2)	1 (1.2)		

## Discussion

The current study aimed to assess the practices of maternity nurses in terms of perinatal bereavement care. The study findings answered the research question. The findings revealed that approximately three-quarters of the studied maternity nurses reported a satisfactory level of practices regarding perinatal bereavement care. In contrast, approximately one quarter reported an unsatisfactory level of practices. These findings contrast with a Malaysian study [15], which reported that more than half of the studied nurses had practiced favourable caring behaviors regarding perinatal bereavement care. This dissimilarity between the studies could be attributed to inadequate training regarding perinatal bereavement care.

Additionally, this study disagrees with a Japanese study [20], which revealed a disparity between the expected care and that actually provided to bereaved families, especially in key services such as counselling, education, peer support, and ongoing family support. Additionally, in contrast with the findings with an Indian study [21], which reported that approximately one-third of participants provided bereavement care following foetal loss. This dissimilarity of the studies’ findings may be related to differences in the studied sample sizes, experiences, and level of education.

According to the results of the present study, over one-third of the nurses who were studied provided accommodations for women who were admitted to the hospital with a diagnosis of perinatal loss, away from other cases of normal pregnancy. This contrasts with a Spanish study [22], which reported that when hospital occupancy was permitted, most of the studied participants reported that an individual room was provided, and approximately half of them allowed admission to nonmaternal and child units. This dissimilarity between the studies’ findings may be due to an overcrowded working department with patients, which made it difficult to specify space for bereaved women.

Moreover, the present study revealed that the majority of the studied nurses provided verbal and nonverbal information regarding diagnosis and treatment sensitively to bereaved women. This finding was incongruent with a Ugandan study [23], which reported that the feeling of inability to inform women about their diagnosis of foetal death dominated most of the responses. This dissimilarity between the studies’ findings may be due to differences in cultural attitudes toward death and bereavement, communication norms about delivering bad news, and institutional protocols for bereavement disclosure. Egyptian cultural and religious context may influence communication approaches differently than western settings.

Furthermore, the results of the present study show that the majority of the studied nurses reported that they requested an obstetric ultrasound to confirm IUFD before the decision of delivery. These findings partially matched with a Kenyan study [17], which reported that most of the studied healthcare providers requested obstetric ultrasonography for confirmation of foetal death. The similarity between the studies’ results may be due to medicolegal constraints in the working environment, which demand confirmation of foetal death before making a termination decision.

Additionally, the findings of the present study revealed that more than three-quarters of the nurses did not ask bereaved women about their preference for the mode of delivery and provided an explanation regarding their preference. This finding contrasts with an Indian study [21], which reported that approximately one-seventh of the participants were never asked bereaved women about their preferred mode of delivery. This dissimilarity between the studies’ findings may be related to variations in local protocols of management and hospital policy.

The findings of the present study revealed that only one-third of the studied nurses let bereaved women give birth separately from other women who carry a live baby into the uterus. This finding diverges from the findings of a Kenyan study [17], which reported that less than one-tenth of the studied participants allowed bereaved women to deliver in a separate room, away from those who carry a live foetus. This finding may be attributed to overcrowding of the delivery unit, which made it difficult to allocate separate space for the delivery of women diagnosed with perinatal loss.

Moreover, the findings of the present study revealed that none of the studied nurses used a partogram during the labor of women who were diagnosed with stillbirth. This contrasted with the recommendations of National Health Service (NHS) England [24]. Basic management principles of labor, such as bladder care, using a partogram, and detecting delayed or interrupted progress, are the same for bereaved women as for women carrying a live foetus [25]. This gap in current practices may be attributed to a lack of awareness of guidelines and recommendations related to the intrapartum care of bereaved women.

Additionally, the findings of the present study showed that the studied nurses reported an unsatisfactory level of practice regarding allowing skin-to-skin contact with a dead baby, as the majority of them did not apply this practice. This finding was in disagreement with a Nigerian study [26], which reported that the participants showed an acceptable level of psychosocial intervention regarding allowing the bereaved couple to view and cuddle their deceased baby. The disagreement between the results of the studies may be due to differences in the hospital’s policies and rules regarding bereavement care.

The results of the present study revealed that over three-quarters of the nurses who studied offered a referral to another healthcare provider, such as a psychologist. This finding was in disagreement with a Spanish study [13], which reported that more than half of the studied participants can support bereaved families through referrals to psychology/mental health during the postnatal period. This dissimilarity between the studies’ results may be related to variations among health care professionals’ understanding of the importance of psychotherapy for such emotionally stressful situations.

The findings of the present study show that more than three-quarters of the studied nurses discussed the potential benefit of delaying conception with bereaved women until severe psychological issues were resolved. This finding disagrees with an American study [27], which revealed that more than half of the studied participants encouraged bereaved women to take some time before becoming pregnant again. This result could be due to nurses’ understanding of both the physical and the psychological impacts of perinatal loss experience on subsequent pregnancies.

Finally, the current study revealed a highly statistically significant association between the working departments of the studied maternity nurses and their practice level (*P* = 0.000). This finding may be related to their frequent exposure to patients experiencing perinatal loss who require specified perinatal bereavement care, which allows nurses to develop and refine their practical skills. This finding disagrees with a Malaysian study [15], which reported that there is no significant association between nurses’ ward specialty and their practice level.

Perinatal bereavement is a global experience. Therefore, perinatal bereavement care is a critical aspect of maternal and newborn health services that requires compassionate, culturally sensitive, and evidence-based support for grieving families. Health care professionals, particularly nurses, play a vital role in delivering such care and must be equipped with proper training and clear guidelines. While this study did not employ a specific theoretical framework, future research could apply models such as Swanson’s Theory of Caring, Worden’s Tasks of Mourning, or the CARES model to design and evaluate theory-guided bereavement care interventions.

## Conclusion

The findings indicate notable gaps in nurses’ practices regarding perinatal bereavement care, especially during the intrapartum period, which highlights the need for structured and continuous training programs to enhance the quality of bereavement care in the nursing field. Improving nurses’ competencies in this area is essential to positively influence the well-being of bereaved families. Action is required to integrate targeted educational interventions within maternity care settings, while future research should include longitudinal evaluations of such interventions and replicate this study in different geographical regions of Egypt to enhance the generalizability of the findings.

### Recommendations

Based on the study findings, the following recommendations are proposed:

Nurses should allocate time within their schedule for attending training sessions and workshops about bereavement care to improve their practices, especially during the intrapartum period, such as using partogram during the delivery of bereaved women as well as those with live foetuses and allowing bereaved parents’ skin-to-skin contact with deceased babies. Nurses should also accommodate bereaved women away from those who carry live foetuses. Additionally, developing policy for bereavement care standards is essential.

### Strength and limitations

This paper discusses a fairly unknown field within Egypt by assessing the practice of maternity nurses in the field of perinatal bereavement care to provide information to inform the future research. It is based on existing guidelines and research, which provides the validity of data collection by expert check and reliability test. The results provide suggestions on the nursing practice and future research in order to improve perinatal bereavement care. Social desirability bias, however, can be introduced the practice of data collection via self-reporting. Also, convenience sampling used in the study, which was at one hospital, might not be generalizable to other hospitals. In order to enhance the generalizability, the future research should utilize random sampling in various settings. More comprehensive validation (testing of construct validity and greater use of expert panels) would be useful in further research.

## Data Availability

The corresponding author can provide the research data utilized and analysed in the current research upon appropriate request.

## Abbreviations

CBC: Complete blood count
IUFD: Intrauterine Foetal Death
MUH: Mansoura University Hospital
NHS: National Health Service
Rh: Rhesus factor
