# Correction: Practices of maternity nurses regarding perinatal bereavement care: a cross-sectional study

**DOI:** 10.1186/s12884-026-09052-x

**Published:** 2026-04-30

**Authors:** Nora Monier Elsaka, Abdelaziz Elrefaeey, Eman Sameh AbdElhay, Samia I Hassan, Hanan Elsayed Mohamed Elsayed

**Affiliations:** 1 Woman’s Health and Midwifery Nursing, Faculty of Nursing, Mansoura University, Mansoura City, Dakahlia Governorate Egypt; 2 Obstetrics and Gynecology, Faculty of Medicine, Mansoura University, Mansoura City, Dakahlia Governorate Egypt; 3 Psychiatric Nursing and Mental Health, Faculty of Nursing, Mansoura University, Mansoura City, Dakahlia Governorate Egypt

**Correction: BMC Pregnancy Childbirth 26**,** 92 (2026)**

**https://doi.org/10.1186/s12884-025-08563-3**

Following publication of the original article [1], the authors identified an error in Table 3, the column headings “Yes” and “No” were inadvertently interchanged. The correct table is given below. The original article has been corrected.

**Table 3:** Distribution of the studied maternity nurses according to their reported practices regarding perinatal bereavement care during the admission period (*n* = 117)

Practices during the admission period	No	Yes
	*n* (%)	*n* (%)
Welcome the bereaved woman to the ward and provide orientation to the room, call bell, and facilities	14 (12.0)	103 (88.0)
Give the bereaved woman a direct admission card with an explanation	23 (19.7)	94 (80.3)
Accommodate a woman who was admitted to the hospital with a diagnosis of perinatal loss, away from other cases of normal pregnancy	74 (63.2)	43 (36.8)
Scheduling the bereaved woman’s appointment to be the first woman seen by the sonographer and obstetrician on that day	53 (45.3)	64 (54.7)
Providing verbal and nonverbal information regarding diagnosis and treatment in a sensitive manner	19 (16.2)	98 (83.8)
Obtaining past obstetric and medical history on admission	22 (18.8)	95 (81.2)
Offering individualized preparation for delivery tailored to women’s needs and health conditions	21 (17.9)	96 (82.1)
Preparing parents for the appearance of a baby after birth	84 (71.8)	33 (28.2)
Requesting an obstetric ultrasound to confirm IUFD before the decision of delivery	15 (12.8)	102 (87.2)
Requesting Rh, CBC, and blood clotting tests on admission	5 (4.3)	112 (95.7)
Asking bereaved women about their preference for the mode of delivery and explaining their preference	91 (77.8)	26 (22.2)
Obtaining written consent for any invasive procedure on the baby, including tissues taken for genetic analysis	40 (34.2)	77 (65.8)
Offering pain relief measures according to hospital policy	75 (64.1)	42 (35.9)
